# Antimicrobial Resistance Profiles and Genes in *Streptococcus uberis* Associated With Bovine Mastitis in Thailand

**DOI:** 10.3389/fvets.2021.705338

**Published:** 2021-08-17

**Authors:** Tingrui Zhang, Guoyi Niu, Sukolrat Boonyayatra, Duangporn Pichpol

**Affiliations:** ^1^Doctor of Philosophy Program in Veterinary Science, Faculty of Veterinary Medicine, Chiang Mai University, Chiang Mai, Thailand; ^2^Department of Food Animal Clinic, Faculty of Veterinary Medicine, Chiang Mai University, Chiang Mai, Thailand; ^3^Research Group for Veterinary Public Health, Faculty of Veterinary Medicine, Chiang Mai University, Chiang Mai, Thailand; ^4^Department of Veterinary Biosciences and Veterinary Public Health, Faculty of Veterinary Medicine, Chiang Mai University, Chiang Mai, Thailand

**Keywords:** *Streptococcus uberis*, bovine mastitis, antimicrobial resistance, antimicrobial resistance gene, intramammary infection

## Abstract

*Streptococcus uberis* is recognized as an environmental mastitis pathogen in dairy cattle. The varied success rate of antibiotic treatment for *S. uberis* intramammary infection may be associated with the antimicrobial resistance (AMR) of these bacteria. This observational study aimed to analyze 228 *S. uberis* strains associated with bovine mastitis in northern Thailand from 2010 to 2017. AMR and AMR genes were determined by the minimum inhibitory concentration (MIC) using a microdilution method and polymerase chain reaction, respectively. The majority of *S. uberis* strains were resistant to tetracycline (187/228, 82.02%), followed by ceftiofur (44/228, 19.30%), and erythromycin (19/228, 8.33%). The MIC50 and MIC90 of ceftiofur in 2017 were 2–4-fold higher than those in 2010 (*P* < 0.01). Resistance to tetracycline and ceftiofur significantly increased between 2010 and 2017 (*P* < 0.05). The most common gene detected in *S. uberis* was *tetM* (199/228, 87.28%), followed by *ermB* (151/228, 66.23 %) and *blaZ* (15/228, 6.58 %). The association between tetracycline resistance and *tetM* detection was statistically significant (*P* < 0.01). The detection rates of *tetM* significantly increased, while the detection rates of *tetO* and *ermB* significantly decreased during 2010–2017. AMR monitoring for bovine mastitis pathogens, especially *S. uberis*, is necessary to understand the trend of AMR among mastitis pathogens, which can help create an AMR stewardship program for dairy farms in Thailand.

## Introduction

Bovine mastitis, usually caused by an intramammary infection (IMI) of microorganisms, contributes to a major loss in dairy production by decreasing the production of milk and early culling of dairy cows (1). *Streptococcus uberis* commonly causes mastitis in dairy cattle worldwide (2). Although the major sources of *S. uberis* in dairy farms include water, soil, plant matter, bedding materials, flies, and hay (2), several studies have suggested that transmission of *S. uberis* can also occur between cows (3, 4).

Clinical mastitis caused by *S. uberis* accounts for approximately 45% of *S. uberis* IMIs during lactation (5). The treatment of bovine mastitis associated with *S. uberis* relies on the use of antimicrobial agents. Various groups of antimicrobial agents, such as macrolides, lincosamides, beta-lactams, and cephalosporins, were reported for the treatment of *S. uberis* IMI (6). However, the bacteriological cure rates following antimicrobial treatment of clinical mastitis caused by *S. uberis* are reported to vary, ranging from 64 to 91% (7–9). The variation in the success of treatment can be attributed to the virulence and antimicrobial resistance (AMR) of the pathogen.

AMR has become a worldwide problem for both human and animal health (10). In the dairy industry, antimicrobial agents have been mainly used for the treatment of bovine mastitis and in dry cow therapy (11, 12). The excessive use of antimicrobial agents in dairy herds may lead to increased AMR among mastitis pathogens (12). Therefore, monitoring AMR trends over a period of time is necessary to create an effective antimicrobial stewardship in dairy herds.

Thailand is a tropical country in Southeast Asia. In 2019, the Department of Livestock Development of Ministry of Agriculture and Cooperatives in Thailand reported the population of dairy cattle in the country to be approximately 670,000 with approximately 300,000 milking cows in 19,000 dairy farms (13). Northern Thailand is one of the dairy-intensive regions. Approximately 83,000 dairy cattle or 12.4% of the total dairy cattle population in the country are in northern Thailand. Bovine mastitis has been a major health problem in dairy cattle in this region (14).

In northern Thailand, *S. uberis* was reported to be a common pathogen associated with clinical and subclinical mastitis (15). Although AMR among *S. uberis* associated with bovine mastitis was reported in many countries (16, 17), reports in Southeast Asia, including Thailand, are limited. We aimed to investigate the AMR phenotypes and genotypes of *S. uberis* isolated from bovine mastitis cases in northern Thailand. The observed AMR patterns may result in an effective treatment protocol for *S. uberis* IMI in this region.

## Materials and Methods

### Sample Selection

**Figure 1:**
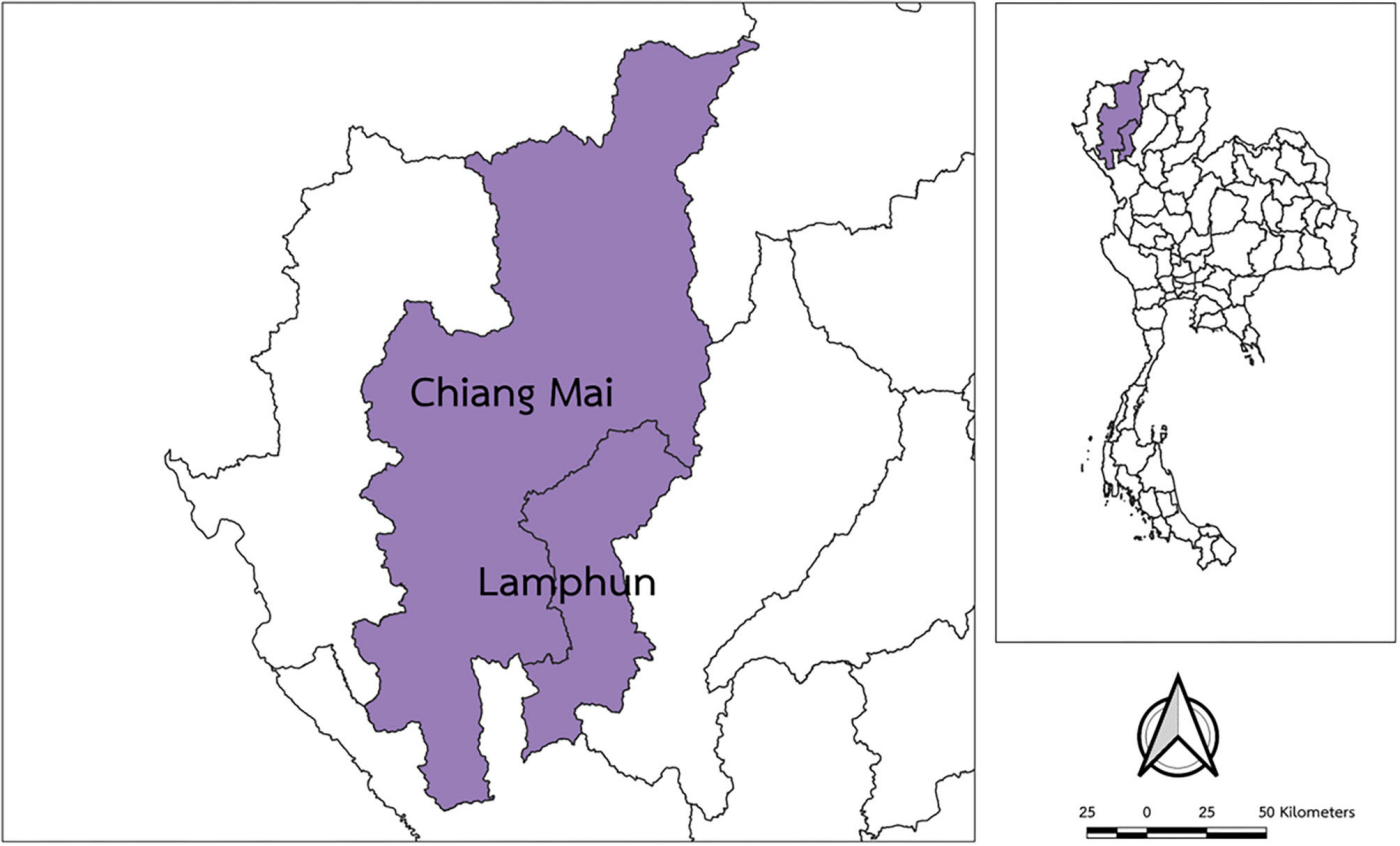
Geographical map of Chiang Mai and Lamphun provinces in northern Thailand.

This is an observational study that investigated phenotypic and genotypic AMR among archived isolates of *S. uberis*. *S. uberis* isolated from milk samples of cows with clinical or subclinical mastitis, submitted to the Faculty of Veterinary Medicine in Chiang Mai University (Thailand) between January 2010 and December 2017, were included in the study. All milk samples were collected and cultured as part of some previous programs during 2010–2017 from dairy cattle herds in Chiang Mai and Lamphun provinces in northern Thailand (Figure 1). These two provinces represent the most dairy-intensive region in northern Thailand, with an approximate population of 34,000 milking cows and 1,500 dairy farms (13). In this region, most dairy cattle are crossbred Holstein and raised in tied stalls. A bucket-type milking system is mostly adopted in this region. Most farms are small-holder dairy farms with 15–60 milking cows producing approximately 12 kg of milk/cow/day.

All milk samples in 2010–2011 were passively submitted for the diagnosis of IMI by local veterinarians. During 2012–2017, not only milk samples passively submitted for the diagnosis of IMI, but also milk samples of cows actively screened for clinical and subclinical mastitis as requested by owners of herds with history of high bulk milk somatic cell count, were the sources of *S. uberis* isolates. The mastitis screening by veterinarians was the service program provided by the university during that particular period. Clinical mastitis cases were considered for cows presenting abnormal milk and/or changes of the udder such as swelling, pain, and heat. Subclinical mastitis cases were diagnosed using the California Mastitis Test. As a part of the veterinary services, quarter milk samples were aseptically collected and submitted for the diagnosis of mastitis pathogens. The *S. uberis* isolates were kept frozen at −80°C in a brain heart infusion broth containing 20% glycerol until use. The sampling criteria for selecting *S. uberis* isolates were different based on the total number of isolates originated each year. For years with a low number of *S. uberis* isolates (*n* ≤ 10), all isolates were included in the study. For years with more than 10 isolates, 1–6 isolates per farm per isolated month were selected in order to include the highest diversity of *S. uberis* isolates in those years. Given these criteria, a sample of 228 isolates from a total of 442 frozen isolates was randomly selected for each month of each year as a representative collection of *S. uberis* isolates in each studied year. These *S. uberis* isolates were from 55 clinical and 173 subclinical cases of 165 cows in 105 farms.

### Identification of *S. uberis* Isolates

All selected frozen *S. uberis* isolates were re-grown on blood agar (Merck®, Darmstadt, Germany) with 5% bovine blood and incubated at 37°C for 24 h. Tests such as Gram staining, catalase test, and the ability to metabolize esculin, inulin, mannitol, and salicin were used to identify *S. uberis*. The genomic DNAs of all isolates were extracted using a DNA extraction kit (NucleoSpin®, Düren, Germany) and confirmed to be *S. uberis* using polymerase chain reaction (PCR) to detect the 16S rRNA gene (Table 1), as described previously (3).

**Table 1:** DNA Sequences, target genes, and expected product sizes of PCR primers used for the identification of *Streptococcus uberis* and detection of antimicrobial resistance genes.

**Target gene**	**Primer**	**Primer sequence (5'−3')**	**Product size (bp)**	**References**
16S rRNA	Forward	CGCATGACAATAGGGTACA	445	Hassan et al. (18)
	Reverse	GCCTTTAACTTCAGACTTATCA		
*pbp2b*	Forward	GATCCTCTAAATGATTCTCAGGTGG	1,500	du Plessis et al. (19)
	Reverse	CAATTAGCTTAGCAATAGGTGTTGG		
*blaZ*	Forward	TTAAAGTCTTACCGAAAGCAG	377	Bagcigil et al. (20)
	Reverse	TAAGAGATTTGCCTATGC		
*tetL*	Forward	TGAACGTCTCATTACCTG	993	Lopardo et al. (21)
	Reverse	ACGAAAGCCCACCTAAAA		
*tetO*	Forward	AACTTAGGCATTCTGGCTCAC	519	Lopardo et al. (21)
	Reverse	TCCCACTGTTCCATATCGTCA		
*tetM*	Forward	GAACTCGAACAAGAGGAAAGC	740	Lopardo et al. (21)
	Reverse	ATGGAAGCCCAGAAAGGAT		
*ermB*	Forward	ATTGGAACAGGTAAAGGGC	442	Marimón et al. (22)
	Reverse	GAACATCTGTGGTATGGCG		
*mefA*	Forward	AGTATCATTAATCACTAGTGC	346	Sutcliffe et al. (23)
	Reverse	TTCTTCTGGTACTAAAAGTGG		
*aac(6′)-Ie-aph(2″)-Ia*,	Forward	GAGCAATAAGGGCATACCAAAAATC	348	Kao et al. (24)
	Reverse	CCGTGCATTTGTCTTAAAAA ACTGG		
*aph(2″)-Ib*	Forward	TATGGATCCATGGTTAACTTGGACGCTGAG	121	Kao et al. (24)
	Reverse	ATTAAGCTTCCTGCTAAAATATAAACATCTCTGCT		
*aph(2″)-Id*	Forward	GG TGGTTTTTACAGGAATGCCATC	642	Kao et al. (24)
	Reverse	CCCTCTTCATACCAATCCATATAACC		

*PCR, polymerase chain reaction*.

### Determination of Antimicrobial Susceptibility of *S. uberis*

All *S. uberis* isolates were investigated for their antimicrobial susceptibility using the microdilution method recommended by the Clinical and Laboratory Standards Institute (CLSI) performance standards (25). Five antimicrobial agents were selected for testing: penicillin G, ceftiofur, erythromycin, tetracycline, and gentamicin. These antimicrobial agents were selected as representative drugs from five antimicrobial classes, namely penicillins (penicillin G), cephalosporins (ceftiofur), macrolides (erythromycin), tetracyclines (tetracycline), and aminoglycosides (gentamicin), which are commonly found in dairy herds. All antimicrobial agents were diluted in Mueller Hinton broth (MHB) according to the selected concentration ranges considered from literature reviews. The selected diluted concentration ranges were 0.0039–4 μg/mL for penicillin G, 0.0625–256 μg/mL for ceftiofur, 0.0039–8 μg/mL for erythromycin, 0.625–64 μg/mL for tetracycline, and 0.0039–16 μg/mL for gentamicin. A single colony of each *S. uberis* isolate was cultured in MHB. The turbidity of the inoculated MHB was adjusted to 0.5 McFarland using a McFarland spectrophotometer. *Streptococcus pneumoniae* ATCC® 49619 and *Escherichia coli* ATCC® 25922 were used as quality control strains. The minimum inhibitory concentration (MIC) breakpoint standards are listed in Table 2. The MICs of each antimicrobial agent that inhibited the visible growth of ≥50 and ≥90% of microorganisms (MIC50 and MIC90) were recorded for each antibiotic.

**Table 2:** Antimicrobial susceptibility breakpoints used to determine the antimicrobial susceptibility (S), intermediate (I), and resistance (R) of *Streptococcus uberis*.

**Antimicrobial**	**Breakpoint (μg/mL)**			**References**
	**S**	**I**	**R**	
Penicillin G	≤ 0.12	0.25–2	≥4	CLSI VET08 ED4 (25)
Gentamycin	≤ 4	8	≥16	CLSI M31-A3 (26)
Erythromycin	≤ 0.25	0.5	≥1	CLSI VET08 ED4 (25)
Tetracycline	≤ 2	4	≥8	CLSI VET08 ED4 (25)
Ceftiofur	≤ 2	4	≥8	CLSI VET08 ED4 (25)

*CLSI, clinical and laboratory standards institute*.

### Detection of AMR Genes

All *S. uberis* isolates were screened for AMR genes using PCR. The screened AMR genes included *blaZ* and *pbp2b* for beta-lactam resistance; *tetL, tetO*, and *tetM* for tetracycline resistance; *ermB* and *mefA* for macrolide resistance; and *aac(6*′*)-Ie-aph(2*″*)-Ia, aph(2*″*)-Ib*, and *aph(2*″*)-Id* for aminoglycoside resistance. The primers used for each gene are listed in Table 1. The PCR mixture (25 μL) contained 0.5 μL of forward and reverse primers (10 mol/L); 12.5 μL of 2X Taq Master Mix containing 1.25 U of Taq DNA polymerase, 1X ViBuffer A, 0.2 mM dNTPs, and 1.5 mM MgCl_2_ (MyTaq™ Red Mix; Bioline, NSW, Australia); 11 μL of DNase-free water; and 0.5 μL of DNA template (50–100 ng/μL). The tubes were placed in a thermal cycler with the following program: initial denaturation at 94°C for 5 min; 35 cycles of denaturation at 94°C for 30 s; annealing temperature as shown in Table 1, for 30 s; and an extension at 72°C for 60 s. The PCR products were determined by 2% agarose gel electrophoresis, stained with ethidium bromide, and visualized under ultraviolet illumination.

### Statistical Analysis

The MIC50 and MIC90 values were descriptively reported for each antimicrobial agent. AMR and distribution of detected AMR genes were expressed as percentages. MICs, AMR, and AMR gene detection were calculated separately for *S. uberis* isolates each year. Temporal trends in the MIC distributions of each antimicrobial agent were analyzed using the proportional-odds cumulative logit model analysis. The trends of AMR and AMR gene detection rates over the 8-year period (2010–2017) were analyzed using the Cochran–Armitage test for trend and logistic regression model analyses, which depicted “year of isolation” as the exposure variable and “AMR” or “AMR gene detection” as the outcome variable. These analyses were based on the principles previously described by Michael et al. (27) and Aerts et al. (28). The associations between AMR and the presence of AMR genes were determined using the χ^2^-test and Fisher's exact test at a significance level of *P* < 0.05. All statistical analyses were performed using the R statistical software version 4.0.0 (29).

## Results

### AMR Determined by MICs

The MIC of each antimicrobial agent among all *the S. uberis* isolates is shown in Table 3. Most examined *S. uberis* isolates were resistant to tetracycline (187/228, 82.02%), followed by ceftiofur (44/228, 19.30%) and erythromycin (19/228, 8.33%). All examined *S. uberis* isolates were susceptible to penicillin G and gentamicin. A total of 53 isolates (23.25%) were considered to show multidrug resistance, which were defined as strains resistant to two or more antimicrobial agents (Table 4). The MIC50 and MIC90 of each antimicrobial agent among *the S. uberis* isolates in each year are shown in Table 5. The MIC50 and MIC90 of ceftiofur in 2017 were 2–8-fold higher than those in 2010, while the MICs of other antimicrobial agents were stable throughout the 8-year period. The proportional odds cumulative logit model analysis indicated a significant increase in MICs of ceftiofur (*P* < 0.001), tetracycline (*P* < 0.05) and penicillin G (*P* < 0.001) from 2010 to 2017 as shown in Table 6.

**Table 3:** Distribution of MIC for *Streptococcus uberis* (*n* = 228) isolates from dairy cattle with subclinical or clinical bovine mastitis in northern Thailand during 2010–2017^a^.

**Antimicrobial agents**	**Number of isolates with a MIC (μg/mL) of**																**Resistance rate (%)**	**MIC50 (μg/mL)**	**MIC90 (μg/mL)**
	**>32**	**32**	**16**	**8**	**4**	**2**	**1**	**0.5**	**0.25**	**0.125**	**0.0625**	**0.03125**	**0.0156**	**0.0078**	**0.0039**	** <0.0039**			
Ceftiofur	3	7	7	27//	92	58	9	2	15	8	0						19.30	4	8
Tetracycline	178	7	0	2//	3	1	7	19	9	2	0						82.02	>32	>32
Erythromycin					16^b^	0	3//	0	1	1	1	0	0	1	0	205	8.33	<0.0039	<0.0039
Gentamycin			0//	0	0	92	75	44	13	2	0	1	1	0	0	0	0	1	2
Penicillin G					0//	0	3	2	2	9	13	27	33	97	0	42	0	0.0156	0.0625

a *The shaded areas indicate the concentrations of the different antimicrobial agents for which the bacterial isolates were not tested. Double slashes indicate the breakpoints for resistance according to the Clinical and Laboratory Standards Institute (2008; 2018)*.

b *Sixteen isolates were observed to grow in all tested dilutions of erythromycin. These isolates were considered to have MIC of >2 μg/mL of erythromycin*.

*MIC, minimum inhibitory concentration*.

**Table 4:** Distribution of antimicrobial resistance patterns among *Streptococcus uberis* (*n* = 228) isolates from dairy cattle with bovine mastitis in northern Thailand during 2010–2017.

**Antimicrobial agents^a^**	**Number**	**Detection rate (%)**
EFT	9	3.95
TET	134	58.77
ERY	0	0.00
GEN	0	0.00
PEN	0	0.00
EFT + TET	34	14.91
TET + ERY	18	7.89
EFT + TET + ERY	1	0.44
No resistance	32	14.04

a *EFT, ceftiofur; TET, tetracycline; ERY, erythromycin; GEN, gentamycin; PEN, penicillin G*.

**Table 5:** Changes in MICs of antimicrobial agents among *Streptococcus uberis* (*n* = 228) isolates from dairy cattle with bovine mastitis in northern Thailand during 2010–2017.

**Antimicrobial agents**	**MIC50/MIC90 (μg/mL)**							
	**2010 (*n* = 9)**	**2011 (*n* = 9)**	**2012 (*n* = 32)**	**2013 (*n* = 17)**	**2014 (*n* = 57)**	**2015 (*n* = 52)**	**2016 (*n* = 18)**	**2017 (*n* = 34)**
Ceftiofur	4/4	2/8	2/4	2/8	4/8	4/8	4/4	8/32
Tetracycline	>32/>32	>32/>32	>32/>32	4/>32	>32/>32	>32/>32	>32/>32	>32/>32
Erythromycin	<0.0039/2	<0.0039/2	<0.0039/ <0.0039	<0.0039/ <0.0039	<0.0039/ <0.0039	<0.0039/ <0.0039	<0.0039/ <0.0039	<0.0039/>2
Gentamycin	1/2	1/2	2/2	1/2	1/2	1/2	1/2	1/2
Penicillin G	0.0625/0.25	0.0078/0.0625	0.0078/0.0078	0.0078/0.0078	0.0078/0.03125	0.0078/0.0625	0.0078/0.03125	0.03125/0.5

*MIC, minimum inhibitory concentration*.

**Table 6:** The five proportional odds cumulative logit models depicting the association between year of isolation and the changes in the minimum inhibitory concentrations (MICs) of five antimicrobial agents among *Streptococcus uberis* (*n* = 228) associated with bovine mastitis in northern Thailand during 2010–2017.

**MICs outcome**	**Coefficients**	**Standard error**	**Odds ratio (OR)**	**95% confidence interval of OR**	***P*-value**
Ceftiofur	0.31	0.07	1.36	1.19–1.55	* <0.001*
Tetracycline	0.21	0.09	1.24	1.04–1.47	*0.014*
Erythromycin	−0.04	0.10	0.96	0.78–1.17	0.663
Gentamicin	−0.01	0.06	0.99	0.88–1.13	0.922
Penicillin G	0.25	0.07	1.29	1.12–1.48	* <0.001*

### Trends of AMR

**Figure 2:**
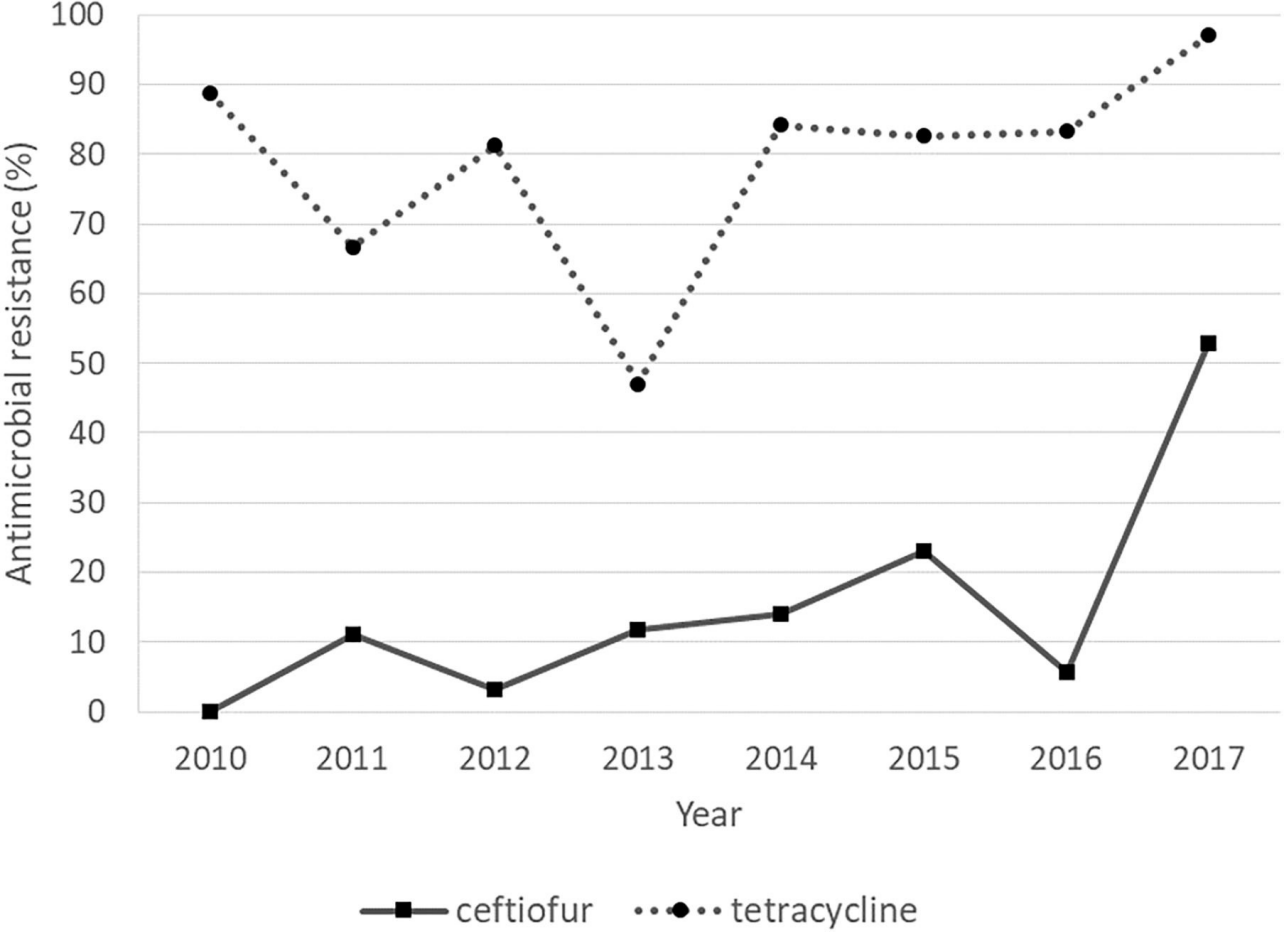
Antimicrobial resistance of *Streptococcus uberis* with “increasing” trends over a period of 8 years, from 2010 to 2017.

The rates of tetracycline resistance ranged from 45 to 100%, which was the highest resistance rate observed in any year. The resistance rate to ceftiofur remained low (<25%) from 2010 to 2016, but it increased to 52.9% in 2017. The resistance rates to erythromycin, gentamicin, and penicillin G were low throughout the study period. Regarding the Cochran–Armitage trend test, the trends of AMR to tetracycline (*P* < 0.05) and ceftiofur (*P* < 0.01) were considered to be “increasing” significantly, showing an increasing resistance during 2010–2017 (Figure 2). The logistic regression model analyses showed similar results, with statistically significant resistance rates to tetracycline (*P* = 0.027) and ceftiofur (*P* < 0.001) as shown in Table 7.

**Table 7:** The three logistic regression models depicting the association between year of isolation and tetracycline-, erythromycin-, and ceftiofur resistance among *Streptococcus uberis* (*n* = 228) associated with bovine mastitis in northern Thailand during 2010–2017.

**Resistance outcome**	**Coefficients**	**Standard error**	**Odds ratio (OR)**	**95% Confidence interval of OR**	***P*-value**
Tetracycline	0.21	0.09	1.23	1.02–1.47	*0.027*
Erythromycin	−0.06	0.13	0.94	0.73–1.20	0.622
Ceftiofur	0.48	0.11	1.61	1.29–2.01	* <0.001*

### Detection of AMR Genes

The distribution of AMR genes detected among *S. uberis* isolates from 2010 to 2017 are shown in Table 8. Among the examined AMR genes, the most commonly detected gene was *tetM* (199/228, 87.28%), followed by *ermB* (151/228, 66.23 %) and *blaZ* (15/228, 6.58 %). The association between tetracycline resistance and *tetM* detection was statistically significant (*P* < 0.01).

**Table 8:** Distribution of antimicrobial resistance gene patterns among *Streptococcus uberis* (*n* = 228) isolates from dairy cattle with bovine mastitis in northern Thailand during 2010–2017.

**Resistance gene**	**Number**	**Detection rate (%)**
*tetM*	57	25.00
*tetL*	0	0.00
*tetO*	0	0.00
*mefA*	1	0.44
*ermB*	13	5.70
*blaZ*	0	0.00
*tetM + ermB*	105	46.05
*tetM + tetL*	2	0.88
*tetO + ermB*	3	1.32
*tetM + blaZ*	3	1.32
*tetM + tetL + blaZ*	1	0.44
*tetM + tetO + ermB*	6	2.63
*tetM + tetL + ermB*	5	2.19
*tetM + mefA + ermB*	6	2.63
*tetM + ermB + blaZ*	11	4.82
*tetM + tetO + mefA + ermB*	1	0.44
*tetM + tetL + tetO + ermB*	1	0.44
No resistance gene	13	5.70

### Trends of AMR Gene Detection Rates

**Figure 3:**
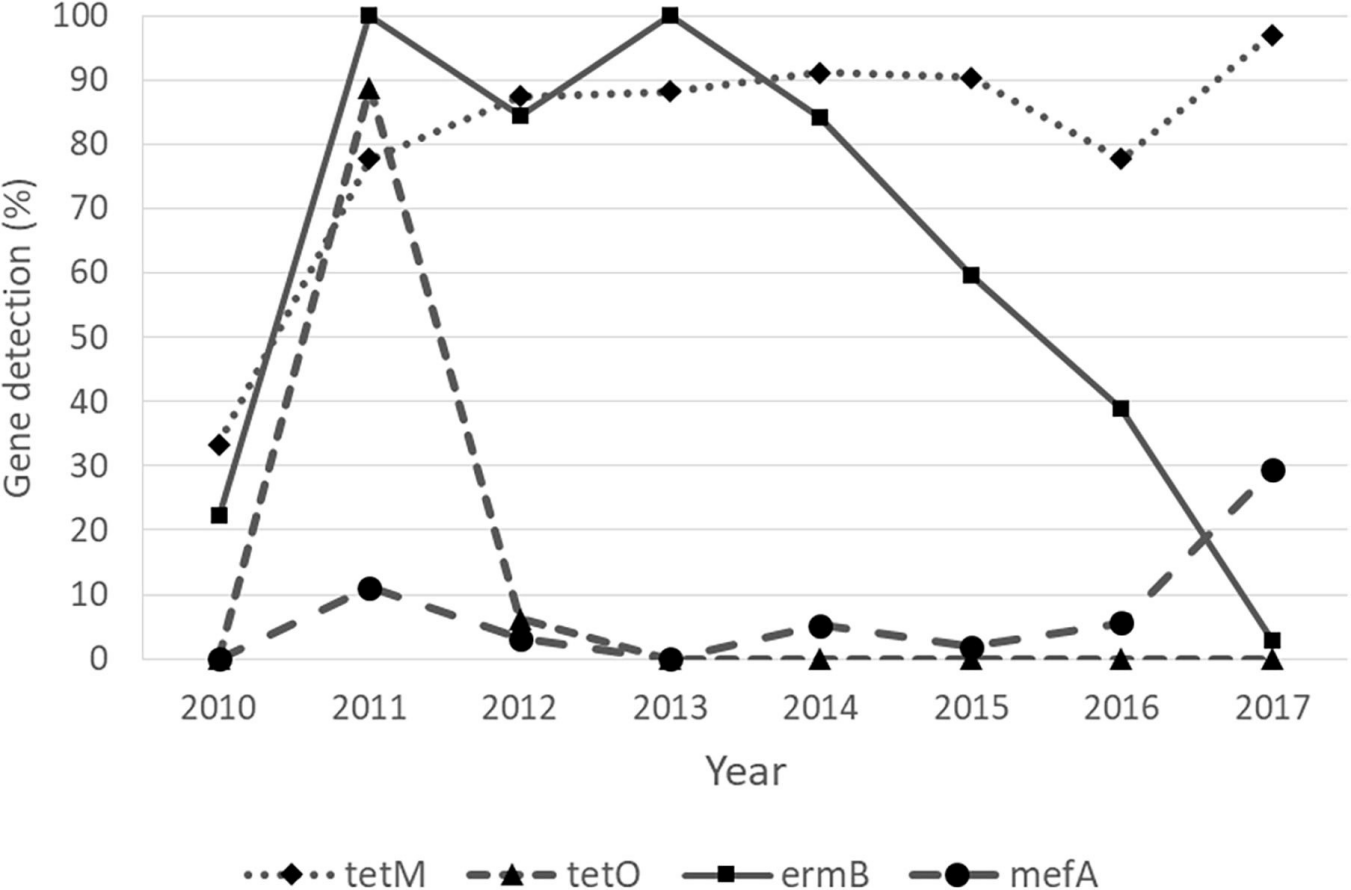
Detection of antimicrobial resistance genes of *Streptococcus uberis* with “increasing” (*tetM* and *mefA*) and “decreasing” (*tetO* and *ermB*) trends over a period of 8 years, from 2010 to 2017.

Regarding the Cochran–Armitage trend test, two genes, *tetM* and *mefA*, showed a significant increase in detection rates from 2010 to 2017 (*P* < 0.01). The detection rates of *tetM* increased from 33.33% in 2010 to 97.06% in 2017, while the detection rates of *mefA* increased from 0% in 2010 to 29.41% in 2017. In contrast, the other two genes, *tetO* and *ermB*, showed a significant decrease in detection rates from 2010 to 2017 (*P* < 0.01). The detection rates of *tetO* were very low throughout the study period, except in 2011, when it peaked at 88.89%. The detection rates of *ermB* were high in 2011–2013 and gradually decreased from 100% in 2013 to 2.94% in 2017. The significant “positive trend” and “negative trend” of AMR gene detection rates are shown in Figure 3. However, when the logistic regression model analyses were performed, only the models of *tetM* (*P* = 0.002)*, tetO* (*P* < 0.001), and *ermB* (*P* < 0.001) detection showed a significant association between the isolated year and detection rates as shown in Table 9.

**Table 9:** The five logistic regression models depicting the association between year of isolation and the detection of *tetL, tetM, tetO, ermB*, and *mefA* among *Streptococcus uberis* (*n* = 228) associated with bovine mastitis in northern Thailand during 2010–2017.

**Detection outcome**	**Coefficients**	**Standard error**	**Odds ratio (OR)**	**95% confidence interval of OR**	***P*-value**
*tetL*	−0.22	0.18	0.80	0.57–1.14	0.218
*tetM*	0.34	0.11	1.40	1.13–1.73	*0.002*
*tetO*	−1.12	0.27	0.33	0.19–0.56	* <0.001*
*ermB*	−0.39	0.09	0.67	0.57–0.80	* <0.001*
*mefA*	−0.02	0.19	0.98	0.67–1.42	0.906

## Discussion

*Streptococcus uberis* is one of the most prevalent environmental pathogens causing bovine mastitis in many regions, including Thailand. *S. uberis* IMI is common during the dry period and early lactation (28). Different classes of antimicrobials were used to treat *S. uberis* IMI (6). The AMR of *S. uberis* can potentially contribute to a successful mastitis treatment.

Our MIC results showed that the *S. uberis* isolates were highly resistant to tetracycline (82.02%) and significantly increased from 2010 to 2017. The tetracycline resistance reported in the current study is higher than that reported in other countries, such as China (59%), Germany (42.3%), Canada (38.6%), and Sweden (12%) (30–33). Tetracycline is widely used in livestock (34); consequently, tetracycline resistance was found to be common in *streptococci* (35, 36). A study in 2016 reported that approximately half of dairy farmers in Chiang Mai province used oxytetracycline for treatment of sick animals in their farms (14). In addition, the antibiotic was slowly eliminated from the body. The slow degradation of tetracycline can lead to an increased selective pressure for tetracycline resistance in bacteria (37). Therefore, although tetracycline is not commonly used to treat mastitis in dairy cattle (38), tetracycline resistance was not unexpected. In the current study, the most frequent tetracycline resistance gene detected was *tetM*, followed by *tetL* and *tetO*. In agreement with the tetracycline resistance, a significant increasing trend of *tetM* detection was observed from 2010 to 2017. In contrast, the trend of *tetO* detection was appeared to be significantly decreased throughout the study period. These findings, together with the significant association between the presence of *tetM* and tetracycline resistance, indicated that tetracycline resistance in *S. uberis* occurs mainly through the functions of the ribosome-protected protein TetM (39). However, a peak of *tetO* detection up to 88.89% in 2011 was observed in the study. This finding could be a result of the limited sample size included in 2010 and 2011. This limited sample size could lead to a less variation of selected isolates, which might consequently affect the detection rates reported in the study.

The current study demonstrated that *S. uberis* isolates were highly susceptible to erythromycin. Similar findings were reported in other countries (32, 40). In northern Thailand, erythromycin and other macrolides, are not commonly used in dairy cattle (14). Therefore, a low erythromycin resistance and low detection rates of erythromycin resistance gene (*ermB*) and macrolide resistance gene (*mefA*) was expected to be observed in this study. However, high detection rates of *ermB* were observed among the *S. uberis* isolates from 2011 to 2013, and gradually decreased from 2013 to 2017. Although previous research suggested that the *ermB* gene dominates erythromycin resistance in *S. uberis* (41), the presence of *ermB* was not associated with the erythromycin resistance among *S. uberis* reported in the current study. Moreover, *mefA* was also detected at a low rate from 2010 to 2016, and increased in 2017. In agreement with the trend of *mefA* detection, MIC90 of erythromycin in 2017 (>2 μg/mL) was observed to be higher than that in 2016 (<0.0039 μg/mL). These findings demonstrated a possible evidence to hypothesize that the *mefA* gene could be responsible for erythromycin resistance among *S. uberis* associated with bovine mastitis in northern Thailand. However, the current study did not reveal a significant association between the presence of *mefA* gene and the erythromycin resistance, which might be caused by the limited sample size with low erythromycin resistance. Therefore, the mechanism of macrolide resistance in *S. uberis* requires further investigation.

Beta-lactam class antimicrobials are frequently used for the prevention and treatment of dairy cattle diseases. In the present study, *S. uberis* showed a high susceptibility to penicillin G (100%) and ceftiofur (81%). This finding is consistent with the results of various studies (31, 36, 40). Likewise, we could not detect the *pbp2b* gene in our sampled *S. uberis*. This gene was reported to play an important role in beta-lactam resistance in streptococci (42, 43). Another gene, *blaZ*, which is responsible for beta-lactam resistance, was also detected at a low level in our *S. uberis* collection, similar to the findings in other previous reports (36, 40).

From 2010 to 2017, we were able to demonstrate an increasing trend of ceftiofur resistance among *S. uberis*, as evidenced by both the resistance rate and MICs from 2016 to 2017. Similarly, a study in China reported an increasing trend of ceftriaxone-resistant strains of *S. dysgalactiae* associated with bovine mastitis (44). Although statistical significance was not observed, an increasing trend was also demonstrated with the MICs of penicillin G. These findings raised the concern of beta-lactam resistance in *S. uberis* in the near future. A study in 2016 reported that more than half of dairy farmers in Chiang Mai province usually used penicillin/streptomycin (52.31%) for systemic treatment of cattle diseases (14). Moreover, most dairy farmers in this region used ampicillin/cloxacillin (87.69%), followed by cephapirin (82.31%) for intramammary treatment of bovine mastitis (14). Because beta-lactam antimicrobial agents are the most commonly used antimicrobial agents for mastitis treatment, the increasing trend of the MICs of these two beta-lactams may provide critically important evidence associated with the cure rate of bovine mastitis caused by *S. uberis* in the future. Antibiotic usage and AMR of *S. uberis* in dairy farms should be continually monitored to adjust the treatment protocols for bovine mastitis in Thailand.

## Conclusions

In conclusion, from 2010 to 2017, *S. uberis* isolates associated with bovine mastitis in Thailand were highly and increasingly resistant to tetracycline, potentially controlled by *tetM*. In addition, throughout the study period, we observed increasing trends in the MICs of ceftiofur, together with an increasing trend in the ceftiofur resistance rate. These findings emphasize the importance of AMR monitoring for bovine mastitis pathogens, especially *S. uberis*, and can serve as guidelines for effective treatment decisions. The prudent use of antimicrobial agents in dairy farms, especially tetracycline and ceftiofur, should be intensively considered and applied in this region. Understanding the trend of AMR among mastitis pathogens can help create an AMR stewardship program for dairy farms in Thailand.

## Data Availability Statement

The raw data supporting the conclusions of this article will be made available by the authors, without undue reservation.

## Author Contributions

TZ, SB, and DP considered the study design. TZ and GN performed the laboratory experiments. SB performed the data analyses. SB and TZ prepared the manuscript. All authors contributed to the article and approved the submitted version.

## Conflict of Interest

The authors declare that the research was conducted in the absence of any commercial or financial relationships that could be construed as a potential conflict of interest.

## Publisher's Note

All claims expressed in this article are solely those of the authors and do not necessarily represent those of their affiliated organizations, or those of the publisher, the editors and the reviewers. Any product that may be evaluated in this article, or claim that may be made by its manufacturer, is not guaranteed or endorsed by the publisher.
